# Pelvic Floor Muscle Training and Its Benefits for Multiple Sclerosis Patients Suffering From Urinary Incontinence and Sexual Dysfunction

**DOI:** 10.7759/cureus.47086

**Published:** 2023-10-15

**Authors:** Vaia Sapouna, Sofia Thanopoulou, Dimitrios Papriakas, Styliani Papakosta, Maria Sakopoulou, Dimitrios Zachariou, Athanasios Zikopoulos, Aris Kaltsas, Nikolaos Vrachnis, Dionysios Vrachnis, Nikolaos Sofikitis, Athanasios Zachariou

**Affiliations:** 1 Physical Therapy Department, Physical Medicine and Rehabilitation Centre Kentavros, Volos, GRC; 2 Physiotherapy Department, University of Thessaly, Lamia, GRC; 3 Physical Medicine and Rehabilitation Department, Physical Medicine and Rehabilitation Centre Kentavros, Volos, GRC; 4 Neurology Department, Physical Medicine and Rehabilitation Centre Kentavros, Volos, GRC; 5 Orthopedic Department, National and Kapodistrian University of Athens, KAT General Hospital, Athens, GRC; 6 Obstetrics and Gynecology Department, Royal Cornwall Hospital, Cornwall, GBR; 7 Urology Department, University of Ioannina, Ioannina, GRC; 8 Obstetrics and Gynecology Department, National and Kapodistrian University of Athens, Attikon Hospital, Athens, GRC; 9 Clinical Therapeutics Department, National and Kapodistrian University of Athens, Alexandra Hospital, Athens, GRC

**Keywords:** quality of life, pelvic floor muscle training, sexual function, urinary incontinence, multiple sclerosis

## Abstract

Several reports have been published during the last decade studying the effect of pelvic floor muscle training (PFMT) in treating urinary incontinence and sexual dysfunction in multiple sclerosis (MS) patients. The aim of the current study is to bring up-to-date findings of earlier systematic reviews, taking into account data published up till June 2023. Databases such as PubMed, Scopus, and EBSCOhost were screened for randomized controlled studies, clinical trials, and systematic reviews. The keywords for the current review were MS, urinary incontinence, sexual function, and PFMT. The implementation of predetermined eligibility criteria permitted an appropriate and convenient study selection. English language publications alone were considered. After removing duplicates and screening the initially recovered articles, an initial search within the present review identified 19 studies. Finally, 10 randomized control trials and two systematic reviews were eligible for evaluation and included in the current review. The outcome measures were the severity of incontinence or overactive bladder, leakage episodes, sexual dysfunction, health-related quality of life, and adherence to PFMT. PFMT is a convenient and effective treatment tool that can significantly improve health-related quality of life and reduce the severity of urinary incontinence and overactive bladder symptoms in people with MS. The present review confirms the effectiveness of specific exercises on leakage episodes, pad usage, sexual dysfunction, compliance to treatment, and treatment satisfaction. Further research is needed to strengthen the reported results.

## Introduction and background

Multiple sclerosis (MS) is a chronic inflammatory pathological condition causing various symptoms that negatively impact the patient’s quality of life. Notably, urinary incontinence affects 19-80% of individuals suffering from MS [1]. While not life-threatening, MS can induce a significant number of negative feelings, including embarrassment, shame, decreased self-esteem, sexual function issues, loneliness, and restricted participation in activities, in the patient [2,3].

Sexual dysfunction appears when the integrity of the sexual response cycle has been damaged, adversely affecting sexual desire, arousal, and functioning [4,5]. Although sexual dysfunction can arise due to various factors, it has been demonstrated that MS is associated with 40-80% of female sexual dysfunction and 50-90% of male sexual dysfunction [6]. It has a detrimental effect on the patient’s quality of life as it generates mood disorders, poor body image, low self-esteem, and emotional challenges in a couple’s life [7].

Pelvic floor muscle training (PFMT) is an effective first-line treatment for various urinary symptoms, including urinary incontinence, bringing about significant improvement [8,9]. Several studies reported enhanced sexual life in non-neurological patient populations [10,11]. Moreover, it is of note that a particular type of training is available for men suffering from urinary incontinence and sexual dysfunction [12,13].

A number of reports have been published over the last decade discussing the effect of PFMT in treating urinary incontinence and sexual dysfunction in MS patients. The present study aims to update the findings of previous systematic reviews, taking especially into account any new research data published up to June 2023.

## Review

Materials and methods

Study Design

This review was developed according to Preferred Reporting Items for Systematic Reviews and Meta-Analyses (PRISMA) guidelines, which is widely recognized as being the most accurate elaboration of a systematic review [14].

Study Inclusion Criteria

A literature search was conducted from 20.7.2023 to 30.7.2023 to produce articles on the examined topics. The inclusion criteria for the review were patients suffering from MS and urinary incontinence and females or males over 18 performing only PFMT for urinary incontinence in one arm of the study. Full-text articles in English, including clinical trials, randomized controlled trials, or systematic reviews, were selected. Articles published in languages other than English or congress abstracts were excluded from the study. There was no search into the grey literature to avoid a possible bias in the study, as reviewers did not evaluate these data.

The primary outcome of the present review is the change in incontinence parameters and sexual function scores from the first assessment to the most recent follow-up evaluation. Secondary outcomes include alterations from baseline to the last available follow-up in the incontinence episodes, number of pads used, pad test, compliance with treatment, and treatment satisfaction.

Search Strategy

The PubMed, Scopus, and EBSCOhost databases were used to conduct the literature search. There were no restrictions on the publication period. MS, urine incontinence, sexual function, and PFMT were the categories of relevant keywords combined to conduct the systematic searches. The keywords are listed in Table 1, and Appendix 1 illustrates the search strategy for each database. The reference list of pertinent articles was also verified again to achieve the best search results. 

**Table 1:** Search MeSH and keywords MeSH, Medical Subject Heading

Category	Search terms
Multiple sclerosis	Multiple sclerosis, chronic progressive, relapsing-remitting, demyelinating diseases, optic neuritis, demyelinating autoimmune diseases - CNS, encephalomyelitis, acute disseminated, myelitis, transverse, chronic progressive multiple sclerosis, progressive relapsing multiple sclerosis, secondary progressive multiple sclerosis, primary progressive multiple sclerosis, relapsing-remitting multiple sclerosis, remitting-relapsing multiple sclerosis, acute relapsing multiple sclerosis, neuromyelitis optica, optic neuritis, Devic disease, demyelinating disease, demyelinating disorder, acute disseminated encephalomyelitis, clinically isolated syndrome, transverse myelitis, acute disseminated encephalomyelitis, encephalomyelitis
Urinary incontinence	Involuntary leakage, involuntary loss, lower urinary tract dysfunction, pelvic floor dysfunction, lower urinary tract symptom, neurogenic bladder dysfunction, neurogenic detrusor overactivity, overactive bladder syndrome, bladder dysfunction, urinary disorders
Sexual function	Sexual function, sexual dysfunction
Pelvic floor muscle training	Electric stimulation therapy, electrical stimulation, magnetic field therapy, magnetic stimulation, urinary catheters, intermittent catheters, pelvic floor muscle training, weighted vaginal cones, biofeedback, scheduled voiding, timed voiding, prompted voiding, lifestyle, lifestyle modification, weight reduction, physical activity, exercise, smoking cessation, smoking reduction, diet, caffeine, fluid manipulation, stress reduction, bladder rehabilitation, pelvic floor rehabilitation

Study Selection Process

All retrieved articles were imported into the Rayyan Application (Qatar Computing Research Institute) Web app and duplicates were removed (https://www.rayyan.ai). The databases described were searched using the keywords "MS and related disorders," "urinary incontinence," "sexual function," and "pelvic floor muscle training, PFMT." We further broadened our search by including synonyms for these phrases, disorders associated with MS, and other therapies as keywords to find any articles that might be relevant to our study. Titles, abstracts, and entire texts were examined for inclusion by two researchers (VS, ST) independently. Those who did not fit the criteria were eliminated. The full texts of all pertinent studies were sought out, downloaded, and assessed as to whether they met the requirements for inclusion. A third reviewer (AZ) was consulted to reach a consensus in the case of discrepancies. The extracted data consisted of author and year of publication, purpose of article, database, sample size, study design, assessment, and reported outcomes.

Results

Flow of Studies

**Figure 1:**
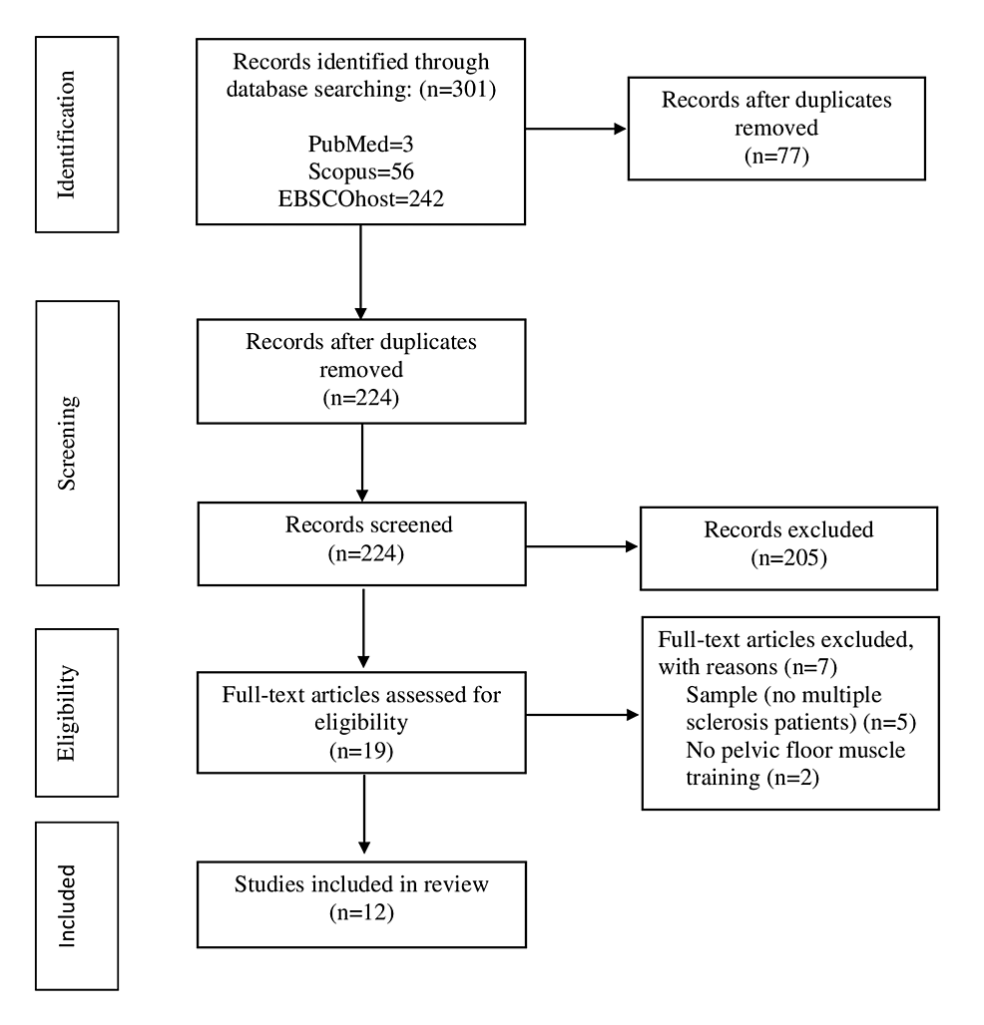
PRISMA flowchart of the selection process PRISMA, Preferred Reporting Items for Systematic Reviews and Meta-Analyses

The initial search from the three electronic databases identified 301 records, of which 77 duplicates were removed. After screening the titles and abstracts of 224 records, 205 were excluded for not meeting the inclusion criteria. Nineteen papers qualified for a second full-text examination for eligibility. Figure 1 provides evidence for the exclusion of seven entries with justifications. A total of 12 studies have been incorporated into this update, including 10 randomized control trials and two systematic reviews.

Study Characteristics and Main Findings

Table 2 describes the characteristics of the reviews, while Table 3 summarizes the characteristics of the clinical trials incorporated in the review. The total number of studies assessed in the two systematic reviews included in our evaluation is 36.

**Table 2:** Characteristics of the reviews LUTD, lower urinary tract dysfunction; MS, multiple sclerosis; NDO, neurogenic detrusor overactivity; PFMT, pelvic floor muscle training; PTNS, peripheral tibial nerve stimulation; WOS, Web of Science

Author and year of publication	Purpose	Databases	Sample size	Study design	Assessment	Reported outcomes
Kajbafval, 2022 [15]	To evaluate the effectiveness of the PFMT as a treatment of LUTD in people with MS	PubMed/Medline, Scopus, PEDro, WOS, CINAHL, Cochrane Library, and Embase	15 studies	Systematic review	Overall effectiveness of PFMT on LUTD in people with MS	PFMT is an effective method to treat both urine leakage and neurogenic bladder symptoms in patients with MS. PFMT positively affects the overall endurance and power of pelvic floor muscles
Vecchio, 2022 [16]	To detect the most effective rehabilitation program for the treatment of bladder disorders in patients with MS and to help physicians in delineating therapeutic tools and programs for physiatrists, to achieve the best clinical results	PubMed, Embase, Cochrane Library, and WOS	21 studies	Systematic review	Efficacy of rehabilitation programs for bladder disorders in patients with MS	Any management of bladder dysfunctions in patients with MS can positively affect the severity of their symptoms and quality of life. Meta-analysis: PTNS and PFMT for NDO constitute two quite effective methods for treating NDO

**Table 3:** The characteristics of the studies comprised in the review AUA, American Urological Association; EDSS, Expanded Disability Status Scale; EMG, electromyography; FSFI, Female Sexual Function Index; HADS, Hospital Anxiety and Depression Scale; HRQoL, health-related quality of life; ICIQ-SF, international consultation on incontinence questionnaire; IIQ, incontinence impact questionnaire; KHQ, King’s health questionnaire; MSQoL-54, multiple sclerosis quality of life; NDS, Neurological Disability Scale; NMES, neuromuscular electrical stimulation; OAB-V8, overactive bladder questionnaire; OABQ-SF, overactive bladder quality of life short-form questionnaire; PFMT, pelvic floor muscle training; SF-36, short-form health survey; UDI, urogenital distress inventory; USP, United States Pharmacopeia

Study and design	Population	Intervention/comparison supervision	Outcomes	Assessment methods
Vahtera, 1997 [17] Open, controlled randomized study in two parallel groups	Control group, n=25, female, age (years)=45.7±10.7, EDSS=4.3 ±2.1, Treatment group, n=25, female, age (years)=42.2±8.9, EDSS=4.4±1.8, Control group, n=15, male, age (years)=41.8±11.8, EDSS=4.4±2.2, Treatment group, n=15, male, age (years)=45.3±.3, EDSS=4.4±1.5	Control group: no treatment; treatment group: electrical stimulation and pelvic floor muscle exercises	Surface EMG, biofeedback, specific questionnaire	Specific questionnaire
McClurg, 2006 [18] Tree-arm randomized controlled trial	Control group, n=10, female, age (years)=49.5±8.7, EDSS=5.4±1.3, Experimental group 1, n=10, female, age (years)=52.1±11.5, EDSS=5.9±1.3, Experimental group 2, n=10, female, age (years)=49.9±11.6, EDSS=5.7±1.0	Control group: Advise+gradual PFMT, 5 times/day × 9 weeks, home-based; Experimental group 1: Advise+Gradual PFMT+Biofeedback, 5 times/day × 9 weeks, home-based; Experimental group 2: Advise+Gradual PFMT+Biofeedback, 5 times/day + NMES 5-30 min/day × 9 weeks; home-based	Leakage episodes, urinary incontinence severity, 24-h pad test, HRQoL compliance follow-up = 0,9,16,24 weeks	3-day bladder diary 24-h pad test, KHQ IIQ UDI MSQoL-54
Khan, 2010 [19] Randomized controlled trial	Whole sample n=58 TG, age (years)=49.7±9.1, CG Age (years)=51.9±9.2	Treatment group: bladder rehabilitation, bladder re-education, behavior management, PFMT, timed and double voiding, intermittent catheterization; Control group: no intervention, regular care	Urogenital distress, neurological disability scale, urinary symptoms severity, incontinence severity	UD16 NDS AUA Symptom Index IIQ7
Lúcio, 2011 [20] Two-arm randomized controlled trial	Control group, n=14, age (years)=34.7±8.8, EDSS=3.3±1.5, Experimental group, n=13, age (years)=36.0±7.2, EDSS=3.4±1.5	Control group: a sham procedure which occurred only on the introduction of a perineometer inside the vagina for 30 min, 2 times/week × 12 weeks, supervised; Experimental group: 30 slow contractions and 3 min of fast contractions with a perineometer 30 min, 2 times/week × 12 weeks, supervised + 30 slow contractions and 3 min of fast contractions, 3 times/day × 12 weeks, home-based	Urge urinary incontinence episodes, overactive bladder severity urinary incontinence severity, HRQOL compliance follow-up = 0,12 weeks	3-day bladder diary OAB-V8 SF-36 Qualiveen ICIQ-SF
Gaspard, 2014 [21] Randomized control trial	Treatment group 1, n=31, age (years)=43.5±14, EDSS=3 for both groups, Treatment group 2, n=31, age (years)=40.5±9.5, EDSS=3 for both groups	Treatment group: 1 PFMT (by using biofeedback); Treatment group: 2 TTNS	Overactive bladder severity follow-up = baseline, after treatments	SF-Qualiveen USP questionnaire 3-day bladder diary
Perez, 2020 [22] Two-arm randomized controlled trial	Control group, n=21, age (years)=47.8±7.24, EDSS=4.8 ± 1.23, Experimental group, n=19, age (years)=45.8±10.5, EDSS=4.8±0.88	Control group: 8-12 slow contractions then an additional 3-4 rapid contractions, three times daily × 12 weeks, home-based; Experimental group: PFMT with the help of internal palpation for 30 min weekly with a physiotherapist × 12 weeks, supervised + 8-12 slow contractions then an additional 3-4 rapid contractions, three times daily × 12 weeks, home-based	Leakages episodes, HRQOL urinary incontinence severity overactive bladder severity compliance follow-up = 0,4,8,12 weeks	3-day bladder diary ICIQ-SF OABQ-SF Exercise diary
Lúcio, 2010 [23] Two-Arm Randomized Controlled Trial	Whole sample n=27, female, Control group, n=NR, age (years)=34.7±8.8, EDSS=3.3±1.5, Experimental group, n=NR, age (years)=36.0±7.2, EDSS=3.4±1.5	Control group: a sham procedure which occurred only on the introduction of a perineometer inside the vagina for 30 min, 2 times/week × 12 weeks, supervised; Experimental group: 30 slow contractions and 3 min of fast contractions with a perineometer 30 min, 2 times/week × 12 weeks, supervised + 30 slow contractions and 3 min of fast contractions, 3 times/day × 12 weeks, home-based	Urge urinary incontinence episodes, pad usage, 24 hr pad test, Overactive bladder severity compliance follow-up = 0,12 weeks	3-day bladder diary OAB-V8 24-hr pad test, Pad usage
Ferreira, 2016 [24] Two-Arm Clinical Trial Whole sample	n=24, female, age (years)=43.3±10.68, EDSS=NR	Control group: 3 sets of 10 contractions, 2 times/week, 48 sessions, home-based; Experimental group: 3 sets of 10 contractions with surface electrostimulation, 2 times/week, 48 sessions, supervised	HRQOL anxiety and depression overactive bladder severity follow-up = baseline, after treatments	Qualiveen HADS OAB-V8
Ferreira, 2019 [25] Two-Arm Clinical Trial	Control group, n=15, age (years)=49.8±16.5, EDSS=3.5±0.8, Experimental group, n=15, age (years)=38.6±13.5, EDSS=3.5±1.0	Control group: 3 sets of 8-10 contractions, twice a week × 6 months, home-based; Experimental group: 3 sets of 8-10 contractions + 20 fast and 20 slow contractions with intravaginal electrostimulation of 30 min, twice a week × 6 months, supervised	HRQOL overactive bladder severity follow-up = baseline, after treatments	Qualiveen OAB-V8
Mosalanejad, 2018 [26] Tree-Arm Randomized Controlled Trial	Control group 1, n=23, age (years)=36.0±7.2, Control group 2, n=23, age (years)=35.7±6.8, Experimental group, n=24, age (years)=35.5±5.7	Control group 1: 2 times/day PFMT (total: 60-100 contractions/per day) × 8 weeks, supervised + home-based; Control group 2: 90 min mindfulness program 1 times/week × 8 weeks, supervised + homework home-based; Experimental group: both mindfulness and PFMT × 8 weeks, supervised + home-based	Sexual function Follow-up = 0,8,12 weeks	FSFI

The systematic review conducted by Kajbafvala et al. evaluated the effectiveness of PMFT as a treatment for lower urinary tract dysfunction (LUTD) in people with MS [15]. After implementing the predefined eligibility criteria, 15 studies were finally included in the systematic review and evaluated as being of moderate to high quality in methodology. According to the results of the present systematic review, PFMT is clearly shown to offer excellent treatment for both urine leakage (standardized mean difference (SMD)=0.50, 95% CI (-0.78, -0.23)) and neurogenic bladder symptoms in patients with MS. The researchers furthermore determined that PFMT exerts a very positive effect on the general endurance and strength of pelvic floor muscles (SMD=1.25, 95% CI (0.69, 1.81), and SMD=0.64, 95% CI (0.24, 1.05), respectively. In this context, the authors concluded that PFMT constitutes a convenient tool for MS patients to manage their urine leakage and neurogenic bladder symptoms and, thus, their quality of life in the short term.

A systematic review and meta-analysis was carried out by Vecchio et al. for the purpose of identifying the most effective rehabilitation program for the treatment of bladder disorders in patients with MS as well as to aid physicians in producing therapeutic tools and programs for physiatrists in the achievement of optimal clinical results [16]. A total of 21 studies met the inclusion and exclusion criteria of the systematic review. The authors, having analyzed the reviewed articles, concluded that the management of bladder dysfunctions in MS patients is capable of substantially improving their symptoms and quality of life. The results show that peripheral tibial nerve stimulation (PTNS) and PFMT effectively treat neurogenic detrusor overactivity (NDO). The authors suggested that a specific therapeutic protocol should be implemented for patients with MS-related neurogenic LUTD based on the severity of their disability and the complexity of their symptoms. This is because the goal of physical therapy is to treat incontinence without exacerbating urinary retention, and vice versa, to reduce urinary urgency while ensuring bladder emptying.

The severity of urinary incontinence was measured in six studies [17-22]. The implementation of PFMT significantly reduced the severity of urinary incontinence symptoms, and the clinical outcomes proved to be even better in cases of supervised training under a physiotherapist. In addition, seven studies made assessments of overactive bladder severity [17,20-25]. Six of the studies demonstrated a significant lessening of symptoms when the patients performed PFMT. Of note, the diminished severity of bladder overactivity was seen to be most successful when electrotherapy was added to the treatment [27]. Another outcome that has been evaluated in four studies is the occurrence of leakage episodes [18,20,22,23]. The results revealed that after treatment with PFMT, the occurrence of leakage episodes was reduced, while the outcomes were better in cases where the patient received biofeedback and neuromuscular electrical stimulation in addition to the PFMT than in the group that received only PFMT [18]. With regard to the 24 h pad test, two of the studies, also included in the systematic review of Yavas et al., demonstrated a substantial reduction in pad weight after the application of PFMT, even better outcomes being achieved when biofeedback and biofeedback plus neuromuscular electrical stimulation were applied in conjunction [18,23,27]. Notably, one study reported a decrease in the usage of pads in MS patients when PFMT was implemented [23].

The effect of PFMT on sexual function in MS patients was evaluated in only one study [26]. Three groups of patients (PFMT, mindfulness, and a combination of these two) were assessed with respect to their sexual function. Improvements in sexual function were reported in all three groups, with no differences between them.

Five articles have assessed compliance with treatment [18,20,22,23]. In two studies, compliance was high, while it was lower than 80% in the other three [17,18,20,22,23]. According to Vahtera et al., most drop-outs were due to the disappearance of urinary tract symptoms or severe relapses in MS [17]. Furthermore, it was reported that a multifaceted and individualized program for bladder rehabilitation reduces disability and improves the quality of life in patients with MS [19].

Seven studies were judged as being of some concern [15,16,18,20,22,25,26]. Two studies by Lúcio et al. and Ferreira et al. were judged as being at high risk of bias [23,24].

All the evaluated studies share a common limitation, which is the relatively short follow-up period, typically not exceeding 24 weeks. However, due to the unpredictable nature of MS and the possibility of changing conditions in the future, conducting comprehensive long-term follow-up studies can be logistically complex and resource-intensive. Nonetheless, addressing this gap in evidence is crucial for gaining a deeper understanding of the lasting effects of treatments and improving the management of MS in the long run.

Discussion

The primary outcome of this review was to compare the effectiveness of PFMT in comparison to placebo, standard care, other conservative treatments, or no treatment in treating urinary incontinence and sexual dysfunction in MS patients. The evidence from 10 studies and two relevant systematic reviews was examined with the aim of confirming or overturning the conclusions of previous systematic reviews. Based on the findings of the present review, the new data seem to confirm that PFMT is a successful treatment option for lowering the impact of urine incontinence and overactive bladder symptoms and increasing health-related quality of life.

A critical evaluation of the findings of the 12 studies included in the current review was carried out. It confirmed that PFMT is a highly suitable tool for MS patients since it is easily applied and results in a significant reduction in the severity of the symptoms of urinary incontinence. The clinical outcomes can, moreover, be further enhanced when the performance of the exercises takes place with the guidance of a professional, i.e., a physiotherapist. The latter has been confirmed by previous studies, such as the article published by Hay-Smith et al., which reported that supervision of PFMT by a physiotherapist improves self-report results [28]. A number of other researchers, e.g., Paiva et al., have reached similar conclusions, stressing the fact that the performance of PFMT is far more effective when conducted with professional supervision, while Price et al. stated exactly the same with special reference to women’s training [29,30]. Finally, Yavas et al. maintained that PFMT carried out under the supervision of a physiotherapist enables the individual to pinpoint his or her personal needs, thus resulting in much better clinical outcomes [27].

Another significant finding of this review is related to the fact that the combined implementation of electrotherapy and PFMT can increase the effectiveness of the latter in lessening the impact of overactive bladder in patients with MS. A study conducted about two decades ago by Wang et al. underlines the important contribution of electrical stimulation, which prevents involuntary contractions by inhibiting detrusor contractions: this is brought about by stimulation of the motor efferent fibers, affecting reflex cycles through the spinal cord [31-33]. According to the results of the aforementioned study, electrical stimulation was superior to PFMT used alone or in combination with biofeedback for the treatment of overactive bladder symptoms.

Regarding the reduction of leakage incidents, PFMT has been shown by almost all the reviewed studies to be quite effective, with the exception of the article by McClurg et al. [18]. Yavas’ explanation, which is based on the duration of treatment, appears rational since firm conclusions cannot be drawn on the basis of a single comparison [27]. On the other hand, other researchers have countered that duration composes a major factor that determines the efficacy of PFMT. According to Yavas et al., the effect of regular PFMT could be initially evaluated within two weeks, followed by a decrease in complaints after two months and improvement six months after the start of the treatment [27]. In addition, Van-Kampen and Geraerts proposed that a 12-week duration of PFMT is essential for patients with a neurological disorder such as a stroke [34]. However, it should be noted that studies included in reviews of the general population showed significant variations in the content and length of the PFMT interventions and a clear exercise prescription could not be reported [10]. As a result, further studies are required to determine the best frequency and duration for a PFMT program [35].

The efficacy of PFMT treatment for urinary leakage and neurogenic bladder symptoms along with its beneficial effect on the general endurance and strength of pelvic floor muscles are all important factors that have been mentioned through the initial systematic review and have been confirmed via the current update [15].

A study in the general population reported that the increase in pad usage time usually causes a decrease in health-related quality of life [36]. Only one study of this review assessed pad usage after the treatment and found a substantial decrease in pad use in the PFMT group compared to the control group [23]. These results suggest that PFMT may considerably lessen the financial burden of the disease, although further study is required.

In addition, examination of the review findings will verify that implementation of PFMT in MS patients can have a highly positive effect on their sexual function. Indeed, Ferreira et al. in their systematic review, underlined the fact that PFMT beneficially affects the sexual life of the general population in a number of ways [24]. Additionally, Shafik et al. have asserted the particular kind of exercises that comprise the PFMT routine positively affect the arousal and orgasm response by strengthening the pelvic floor muscles and increasing involuntary contractions through increasing their strength [37]. Considering the information presented above, one could make the case that regardless of the inclusion of extra techniques, such as biofeedback, PFMT appears to be a tried and tested method for enhancing sexual functioning.

As reported by Pottgen et al. urinary incontinence symptoms experienced by people with MS deteriorate and negatively impact the quality of life, affecting daily living, social life, ability to travel, partner and family relationships, sexual life, and people’s emotional states [7]. It is thus evident that PFMT, by lessening the above symptoms, is able to bring about significant improvement in these people’s quality of life, decreasing the necessity for undue planning, restriction of physical activities, avoidance of social pleasures, shame, low self-esteem, and so on. The two articles included in the current review confirm all the above [15,16].

Data originating from current research suggest that urinary incontinence among women can cause high levels of depression and anxiety, the levels rising further if the severity of urinary incontinence itself rises [38,39]. Although we currently lack a sufficient number of studies to reach clear conclusions, it appears evident that PFMT, whether performed with or without electrotherapy, can positively improve the abovementioned anxiety and depression among MS patients who suffer from urinary incontinence [27].

Engaging in physical activity is a type of behavior, and PFMT can be classified as a form of exercise behavior [40]. Providing support is essential for the behavioral aspects of exercise, often accomplished through supervised guidance during the exercise routine. Individuals with MS who perform PFMT under the supervision of a physiotherapist tend to exhibit greater adherence to the exercise regimen compared to those who perform PFMT unsupervised. Although the currently available evidence lacks a clear conclusion, it is possible to propose that participating in PFMT under the guidance of a physiotherapist is likely to be more effective in inducing behavioral changes and promoting better adherence to the exercise routine. 

In the studies conducted by Lucio et al., participants with MS received home exercises in addition to supervised PFMT, whereas the control group did not engage in home exercise [20,23]. Consequently, the control group had significantly fewer sessions compared to the experimental group, leading to challenges for the researchers in drawing reliable conclusions. While these studies were rated as having relatively robust methodological quality when compared to other studies, the substantial difference in training intensity favoring the experimental group and the presence of concerns related to bias introduce complexities in interpreting the outcomes.

Limitations

The present review has certain limitations. Even though our research included three different scientific databases, the research strategy was jeopardized by the exclusion of other databases that could have brought more studies to light. Additionally, in order to examine as many articles as possible, we did not use inclusion or exclusion criteria based on outcome measures, which led to a significant prevalence of heterogeneity. Further limitations include the exclusion of research published in languages other than English and the inclusion of controlled clinical trials that were not randomized. The latter limitation may have resulted in the unintentional exclusion of a number of other related studies pertinent to the aim of this review.

## Conclusions

The available evidence indicates that PFMT (preferably with supervision or combined with biofeedback) constitutes a convenient and effective treatment tool that can significantly improve an individual’s health-related quality of life and lessen the effects of urinary incontinence and overactive bladder symptoms in patients suffering from MS. We observed improvements in sexual function in both male and female MS patients following PFMT, and these improvements did not significantly differ between them. Our systematic review found some evidence supporting the effectiveness of a particular type of exercise in reducing leakage episodes, pad usage, and improving sexual function. Nevertheless, there is a need for more studies to draw a definitive conclusion. The studies/articles included in this systematic review support the findings of a previous systematic review concerning the decrease in urinary leakage incidents and the enhancement of patients’ quality of life by means of the implementation of PFMT.

## Appendices

Appendix 1: search strategy for each database

PubMed Search Formula via NLM. Results: 3

("multiple sclerosis"[Text Word] OR "chronic progressive"[Text Word] OR "relapsing-remitting"[Text Word] OR "demyelinating diseases"[Text Word] OR "optic neuritis"[Text Word] OR "demyelinating autoimmune diseases cns"[Text Word] OR "encephalomyelitis"[Text Word] OR "acute disseminated"[Text Word] OR "myelitis"[Text Word] OR "transverse"[Text Word] OR "chronic progressive multiple sclerosis"[Text Word] OR "progressive relapsing multiple sclerosis"[Text Word] OR "secondary progressive multiple sclerosis"[Text Word] OR "primary progressive multiple sclerosis"[Text Word] OR "relapsing remitting multiple sclerosis"[Text Word] OR "remitting-relapsing multiple sclerosis"[Text Word] OR "acute relapsing multiple sclerosis"[Text Word] OR "neuromyelitis optica"[Text Word] OR "optic neuritis"[Text Word] OR "devic disease"[Text Word] OR "demyelinating disease"[Text Word] OR "demyelinating disorder"[Text Word] OR "adem"[Text Word] OR "clinically isolated syndrome"[Text Word] OR "transverse myelitis"[Text Word] OR "acute disseminated encephalomyelitis"[Text Word] OR "encephalomyelitis"[Text Word]) AND ("involuntary leakage"[Text Word] OR "involuntary loss"[Text Word] OR "lower urinary tract dysfunction"[Text Word] OR "pelvic floor dysfunction"[Text Word] OR "lower urinary tract symptom"[Text Word] OR "neurogenic bladder dysfunction"[Text Word] OR "neurogenic detrusor overactivity"[Text Word] OR "overactive bladder syndrome"[Text Word] OR "bladder dysfunction"[Text Word] OR "urinary disorders"[Text Word]) AND ("sexual function"[Text Word] OR "sexual dysfunction"[Text Word]) AND ("electric stimulation therapy"[Text Word] OR "electrical stimulation"[Text Word] OR "magnetic field therapy"[Text Word] OR "magnetic stimulation"[Text Word] OR "urinary catheters"[Text Word] OR "intermittent catheters"[Text Word] OR "pelvic floor muscle training"[Text Word] OR "weighted vaginal cones"[Text Word] OR "biofeedback"[Text Word] OR "scheduled voiding"[Text Word] OR "timed voiding"[Text Word] OR "prompted voiding"[Text Word] OR "lifestyle"[Text Word] OR "lifestyle modification"[Text Word] OR "weight reduction"[Text Word] OR "physical activity"[Text Word] OR "exercise"[Text Word] OR "smoking cessation"[Text Word] OR "smoking reduction"[Text Word] OR "diet"[Text Word] OR "caffeine"[Text Word] OR "fluid manipulation"[Text Word] OR "stress reduction"[Text Word] OR "bladder rehabilitation"[Text Word] OR "pelvic floor rehabilitation"[Text Word])

Scopus Search Formula via ELSEVIER Results: 56

( "multiple sclerosis" OR "chronic progressive" OR "relapsing-remitting" OR "demyelinating diseases" OR "optic neuritis" OR "demyelinating autoimmune diseases, CNS" OR "encephalomyelitis" OR "acute disseminated" OR "myelitis" OR "transverse" OR "chronic progressive multiple sclerosis" OR "progressive relapsing multiple sclerosis" OR "secondary progressive multiple sclerosis" OR "primary progressive multiple sclerosis" OR "relapsing remitting multiple sclerosis" OR "remitting-relapsing multiple sclerosis" OR "acute relapsing multiple sclerosis" OR "neuromyelitis optica" OR "optic neuritis" OR "devic disease" OR "demyelinating disease" OR "demyelinating disorder" OR "adem" OR "clinically isolated syndrome" OR "transverse myelitis" OR "acute disseminated encephalomyelitis" OR "encephalomyelitis" ) AND ( "involuntary leakage" OR "involuntary loss" OR "lower urinary tract dysfunction" OR "pelvic floor dysfunction" OR "lower urinary tract symptom" OR "neurogenic bladder dysfunction" OR "neurogenic detrusor overactivity" OR "overactive bladder syndrome" OR "bladder dysfunction" OR "urinary disorders" ) AND ( "sexual function" OR "sexual dysfunction" ) AND ( "electric stimulation therapy" OR "electrical stimulation" OR "magnetic field therapy" OR "magnetic stimulation" OR "urinary catheters" OR "intermittent catheters" OR "pelvic floor muscle training" OR "weighted vaginal cones" OR "biofeedback" OR "scheduled voiding" OR "timed voiding" OR "prompted voiding" OR "lifestyle" OR "lifestyle modification" OR "weight reduction" OR "physical activity" OR "exercise" OR "smoking cessation" OR "smoking reduction" OR "diet" OR "caffeine" OR "fluid manipulation" OR "stress reduction" OR "bladder rehabilitation" OR "pelvic floor rehabilitation" ) AND ( LIMIT-TO ( PUBYEAR , 2021 ) )

EBSCOhost Search Formula Results: 242

( (“multiple sclerosis” OR “chronic progressive” OR “relapsing-remitting” OR “demyelinating diseases” OR “optic neuritis” OR “demyelinating autoimmune diseases, CNS” OR “encephalomyelitis” OR “acute disseminated” OR “myelitis” OR “transverse” OR “chronic progressive multiple sclerosis” OR “progressive relapsing multiple sclerosis” OR “secondary progressive multiple sclerosis” OR “primary progressive multiple sclerosis” OR “relapsing remitting multiple sclerosis” OR “remitting-relapsing multiple sclerosis” OR “acute relapsing multiple sclerosis” OR “neuromyelitis optica” OR “optic neuritis” OR “devic disease” OR “demyelinating disease” OR “demyelinating disorder” OR “adem” OR “clinically isolated syndrome” OR “transverse myelitis” OR “acute disseminated encephalomyelitis” OR “encephalomyelitis”) ) AND ( (“involuntary leakage” OR “involuntary loss” OR “lower urinary tract dysfunction” OR “pelvic floor dysfunction” OR “lower urinary tract symptom” OR “neurogenic bladder dysfunction” OR “neurogenic detrusor overactivity” OR “overactive bladder syndrome” OR “bladder dysfunction” OR “urinary disorders”) ) AND ( (“sexual function” OR “sexual dysfunction”) ) AND ( (“electric stimulation therapy” OR “electrical stimulation” OR “magnetic field therapy” OR “magnetic stimulation” OR “urinary catheters” OR “intermittent catheters” OR “pelvic floor muscle training” OR “weighted vaginal cones” OR “biofeedback” OR “scheduled voiding” OR “timed voiding” OR “prompted voiding” OR “lifestyle” OR “lifestyle modification” OR “weight reduction” OR “physical activity” OR “exercise” OR “smoking cessation” OR “smoking reduction” OR “diet” OR “caffeine” OR “fluid manipulation” OR “stress reduction” OR “bladder rehabilitation” OR “pelvic floor rehabilitation”) )
